# Pulse and CW EPR Oximetry Using Oxychip in Gemcitabine-Treated Murine Pancreatic Tumors

**DOI:** 10.1007/s11307-023-01859-w

**Published:** 2023-10-02

**Authors:** Gabriela Dziurman, Agnieszka Drzał, Aleksandra Anna Murzyn, Maciej Mikolaj Kmiec, Martyna Elas, Martyna Krzykawska-Serda

**Affiliations:** 1 Department of Biophysics and Cancer Biology, Faculty of Biochemistry, Biophysics and Biotechnology Jagiellonian University, 7 Gronostajowa St., 30-387, Krakow, Poland; 2 Department of Radiology, Geisel School of Medicine, Dartmouth College, 1 Rope Ferry Rd, Hanover, NH 03755 USA; 3 Department of Radiation & Cellular Oncology, The University of Chicago, 5758 S Maryland Ave, Chicago, IL 60637 USA

**Keywords:** Mouse pancreatic tumor model, Gemcitabine treatment, Hypoxia, Oximetry, EPR, Oxychip

## Abstract

**Purpose:**

The goal of this work was to compare pO_2_ measured using both continuous wave (CW) and pulse electron paramagnetic resonance (EPR) spectroscopy. The Oxychip particle spin probe enabled longitudinal monitoring of pO_2_ in murine pancreatic tumor treated with gemcitabine during the course of therapy.

**Procedures:**

Pancreatic PanO2 tumors were growing in the syngeneic mice, in the leg. Five doses of saline in control animals or gemcitabine were administered every 3 days, and pO_2_ was measured after each dose at several time points. Oxygen partial pressure was determined from the linewidth of the CW EPR signal (Bruker E540L) or from the T_2_ measured using the electron spin echo sequence (Jiva-25™).

**Results:**

The oxygen sensitivity was determined from a calibration curve as 6.1 mG/mm Hg in CW EPR and 68.5 ms^−1^/mm Hg in pulse EPR. A slight increase in pO_2_ of up to 20 mm Hg was observed after the third dose of gemcitabine compared to the control. The maximum delta pO_2_ during the therapy correlated with better survival.

**Conclusions:**

Both techniques offer fast and reliable oximetry in vivo, allowing to follow the effects of pharmaceutic intervention.

**Supplementary Information:**

The online version contains supplementary material available at 10.1007/s11307-023-01859-w.

## Introduction

Low tissue oxygenation and hypoxic conditions, that develop during growth of solid tumors, favor the formation of an aggressive tumor phenotype, which has a negative impact on almost all anticancer therapies used to date [1]. Therefore, monitoring and imaging of the distribution and variation of oxygen concentrations over time are very important for diagnostic and treatment purposes in biology and medicine.

Measurements of oxygen partial pressure (pO_2_), especially the ability to study pO_2_ changes in real time in the preclinical model, could significantly impact clinical research.

Pancreatic ductal adenocarcinoma (PDAC) tumors are known to be highly fibrotic, desmoplastic, with little vascular network and persistent hypoxia. This unique tumor microenvironment (TME) facilitates disease progression by supporting tumor cell proliferation, metabolic reprogramming, suppressing antitumor immunity, inducing metastasis, and developing therapeutic resistance of PDAC [2, 3].

The Pan_O2 murine model is characterized by high malignancy, poor differentiation, and poor survival, and rapid progression simulates human pancreatic cancer and is considered an appropriate syngeneic model to study the tumor microenvironment [4].

Electron paramagnetic resonance oxygen imaging (EPROI) is a direct method of mapping pO_2_ in preclinical tumors. Most of its applications are performed using soluble spin probes, such as nitroxides or the trityl derivative OX063 or its deuterated analog OX071 [5]. Trityl-based oximetry relies on the linear relationship between pO_2_ and spin–lattice and spin–spin relaxation rates (R_1_ and R_2_) of a liquid probe [6]. Another option, particularly suitable for repeated long-term oxygen measurements, are solid-state probes, such as LiPc, or carbon derivatives [7]. The use of a LiPc crystalline probe as an oxygen sensitive EPR probe was first reported in the early 1990s [8]. Other lithium derivatives have been tested, and LiNc-BuO was chosen because of its high spin density, oxygen sensitivity in a wide range, and resistance to many chemical and physical factors [9, 10]. LiNc-BuO in PDMS, denoted Oxychip, has been used in many animal models and in patients to study oxygenation in the skin and tumors [11–14]. We have decided to use an Oxychip implanted in tumor tissue for non-invasive, fast, precise, and repeatable oximetry during therapy. Molecular oxygen interactions with Oxychip are suitable for measurements using both continuous wave (CW) and pulse EPR resulting in the change in spin probe relaxation times that are also reflected in changes in the linewidth of the Oxychip EPR signal.

Gemcitabine is the standard chemotherapy drug for the treatment of PDAC. It is a cytostatic drug from the group of pyrimidine antimetabolites and an analog of 2′-deoxycytidine—one of the four nucleosides forming part of DNA. Once gemcitabine enters the cell, it undergoes a triple phosphorylation process, which then leads to its incorporation into new DNA in place of 2-deoxycytidine. In relation to 2-deoxycytidine, gemcitabine instead of 2 hydrogen atoms within the C2′ carbon has 2 fluorine atoms, which prevents further bonding of nitrogenous bases, thereby causing cell death [15].

The purpose of the study was to explore the potential of preclinical oximetry using Oxychip in pulse EPR compared to CW EPR and to determine whether PDAC tumor oxygenation before and during chemotherapy correlates with tumor response to gemcitabine treatment.

## Material and Methods

### Animals and Tumor Line

PanO2 murine pancreatic cancer cells were cultured using RPMI 1640 medium with heat-inactivated fetal bovine serum (10% content), trypsin 0.25%, and PBS without Mg^2+^ and Ca^2+^ ions, pH 7,41. Pan_02 a murine pancreatic adenocarcinoma (PDAC) was purchased from DCTD Repository at Frederic National Laboratory for Cancer Research. (MD, USA) by Silesian University (in 2018).

The suspension of 1 million of cells in 20 μl of PBS was injected intradermally (using a 29G needle) into the right hind leg of 12–16 weeks old C57BL/6 male mice (*N* = 29) obtained from the animal breeding facility at the Faculty of Biochemistry, Biophysics and Biotechnology of Jagiellonian University (Cracow, Poland). All experimental procedures were approved by the First Local Ethics Committee of Cracow (Permission No. 151/2022) and all applicable institutional and/or national guidelines for the care and use of animals were followed. The mice were housed under standard laboratory conditions LD:12/12, humidity: 60%, temperature: 23 °C. The standard chow diet with free access to drinking water was provided in community cages.

Observations in the mice were carried out every day from the date of tumor implantation. When the tumor biggest diameter reached about 6 mm, tumor size was estimated using an electronic caliper (with an accuracy of 0.01 mm) in three dimensions. Tumor volume (mm^3^) was calculated using the ellipsoid volume formula: $V=\frac{\pi }{6} abc$, where a, b, and c are the mutual perpendicular diameters.

Mice were randomly assigned to 2 groups: treated with intravenous injection of 60 mg/kg BW gemcitabine (*N* = 14) and control (injected intravenously with saline, *N* = 15). Mice were given therapy every 3 days: on days 0, 3, 6, 9, and 12. The total treatment time was 15 days (5 doses with a 72-h time period after each dose was included), followed by observation time (up to 21 days total).

Experimental endpoint criteria for animal survival were tumor bigger than 350 μL or/and 21 days post first gemcitabine dose and/or 20% loss of initial body weight.

**Fig. 1:**
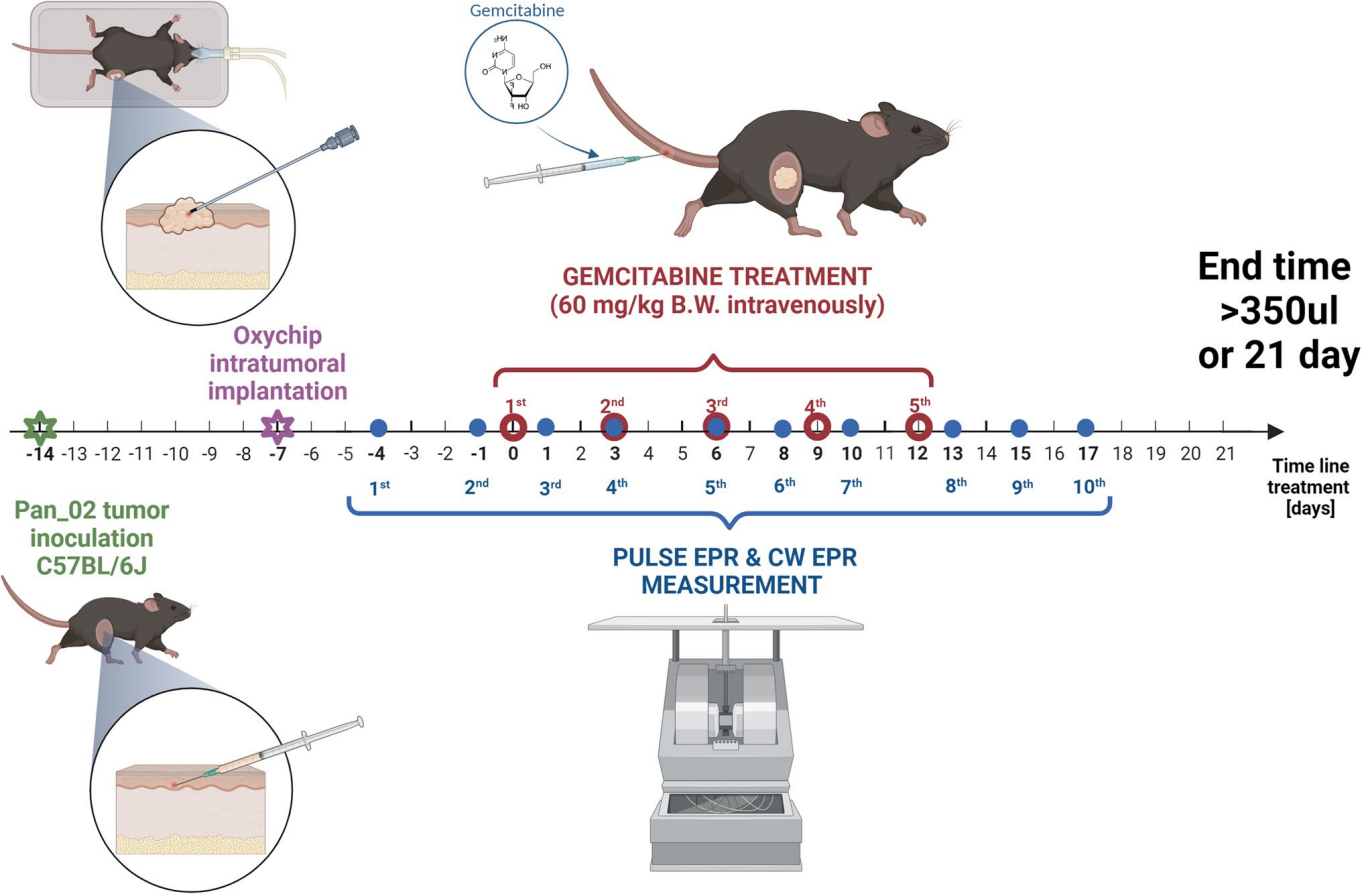
Experimental scheme

The scheme of the experiment, including the timeline, is presented in Fig. 1.

### Calibration Curve

A small piece (1 mm × 1 mm) of Oxychip submerged in 1 ml of saline was used to prepare the calibration curves. All measurements were made at 37 °C. The oxygen calibration curves were constructed using the R_2_ relaxation rate (inverse of the T_2_ relaxation time, pulse measurements) and peak-to-peak linewidths of the first derivative EPR spectra (continuous wave measurements) of the samples measured at different oxygen partial pressures (pO_2_). Pulse EPR measurements were performed using a preclinical oxygen imager JIVA-25™ (O2M Technologies, Chicago, USA) operating at radio frequencies of 685–735 MHz. The settings of the T_2_ relaxation time sequence were as follows: pulse length 60 ns, 8 phase cycles scheme with suppression of FID, repetition time 28 ms, logarithmically spaced delays from 280 ns to 13 μs. Continuous wave spectroscopic measurements were performed on an EPR spectrometer for small animals Bruker Elexsys–II E540L (Bruker Biospin, Germany), operating in the L band (1.1 GHz) with the use of a surface coil and the following parameters: center field 389.1 G, sweep width 10.14 G, modulation frequency 100 kHz, modulation amplitude 0.07 G (occasionally 0.3 G), microwave power 10.75 mW.

Measurements were made in solutions containing 0% (solution deoxygenated with argon), 1.5%, 2.25%, 3%, 4.5%, and 6% oxygen. To achieve the final desired equilibrium oxygen concentration between 0 and 6%, the probe was bubbled in the gas mixture for 30–60 min and then placed in spectrometers. For each oxygen concentration, four to six separate measurements were performed.

### EPR Measurements In Vivo

Once the tumors reached around 3 mm, small fragments of lithium octa-n-butoxynaphthalocyanine (LiNc-BuO) crystals embedded in polydimethylsiloxane (Oxychip was from the lab of Dr. Perianian Kuppusamy, Darmouth College, Hanover, NH, USA [16, 17]) used as a paramagnetic probe were inoculated (using a 18G needle) into tumors. *In vivo* oxygen measurements began 72 h after Oxychip implantation and have been performed twice or three times a week since then. All animals were subjected to inhalation anesthesia using 1–3% isoflurane (Aerrane, Baxter Polska Sp. z o. o., Poland) in the air. The anesthesia depth was controlled carefully by a respiratory pillow sensor (JIVA-25^TM^), so that the breathing rate was not lower than 80 bpm and usually around 100 bpm. The mice were placed in an animal bed and immobilized with vinyl polysiloxane (VPS) dental clay (3M ESPE, USA). Animal’s temperature (37 °C ± 1 °C) was monitored with surface thermometer attached to the mouse’s skin. Pulse EPR was performed using the JIVA-25™ preclinical oxygen imager (O2M Technologies, Chicago, USA) operating at radio frequencies of 685–735 MHz with offset coils for 3D imaging and relaxation times analysis. A horizontal resonator with a dimension of 19 mm × 15 mm was used. The T_2_ relaxation time sequence was used to measure oxygenation with pulse lengths of 60 ns (the same as described above for the calibration curve).

After pulse EPR, mice were transferred to a continuous wave (CW) EPR spectrometer for small animals Bruker Elexsys–II E540L (Bruker Biospin, Germany), operating in the L band. Measurements were made using a surface coil with the following parameters: center field 389.1 G, sweep width 10.14 G, modulation frequency 100 kHz, modulation amplitude 0.07 G (occasionally 0.3 G), microwave power 10.75 mW.

### Ultrasound Imaging

An ultrasound system for preclinical imaging S-Sharp (Scintica, Canada) designed for the examination of small animals with PB406e—Center Frequency 40 MHz (20–50MHz) transducer was used to perform ultrasound imaging. During imaging, the body temperature of the mice was controlled by a heating pad and kept at 37 °C. Anesthesia was induced by 3% isoflurane (Aerrane, Baxter Polska Sp. z o. o., Poland) and then maintained at 1–2.0%.

2D measurements were performed in the B mode (tumor morphology, transducer frequency—50 MHz) and in the Power Doppler mode (tumor vasculature, transducer frequency—40 MHz, Doppler gate size—32 dB).

### Data Analysis

Calculation of T_2_ relaxation time was performed by fitting raw data with function y = a × exp(-2 × tau/T_2_), where tau is delay between pulses. Fitting was done in a Matlab environment with KAZAN data viewer and plugins version 1.4.9.

The analysis of spectra obtained from CW experiments, both during calibration curve preparation and *in vivo* measurements, was carried out using scripts written in a Matlab environment. The first step was a determination of Lorentzian linewidth for deoxygenated Oxychip in saline from the T_2_ relaxation time measured with JIVA-25™. Then, CW spectrum of the same sample was fitted with fixed Lorentzian part to obtain inhomogeneous Gaussian broadening that was later fixed for all fitting procedures. EPR lines were fitted to the probe spectra using the esfit function (part of the EasySpin spectroscopic data analysis package, https://www.easyspin. org/) using the least squares method, the particle swarm algorithm, and first-order perturbation theory. Moreover, the effect of field modulation was included during fitting to extract correct Lorentzian linewidth from our overmodulated data. This procedure eliminates respiratory artifacts and allows accurate oxygen-dependent Lorentzian line width measurements. After fitting, the obtained spectra were interpolated to exclude the impact of spectrum sampling.

Linewidth and R_2_ relaxation rate values obtained from the calibration experiments were plotted against the oxygen concentrations used to construct the calibration curve. The slope of the calibration curve, which is equal to the oxygen sensitivity, was estimated by fitting a straight line to the data points. The linear relationship between line width/R_2_ relaxation rate and oxygen concentration was used to calculate the partial oxygen pressure in tumors measured *in vivo*.

### Statistical Analysis

Statistica13.3® software was used to analyze data with multifactorial and repeated measurements ANOVA to determine the significant effects of factors such as the treatment group or the time scale. For pair analysis, Kruskal-Wallis ANOVA was used. A statistically significant value was considered to be *p* < 0.05. Kaplan-Meier tests were performed to carry out the survival analysis. Linear fits were performed using Origin Pro 2022.

## Results

### Calibration Curves

**Fig. 2:**
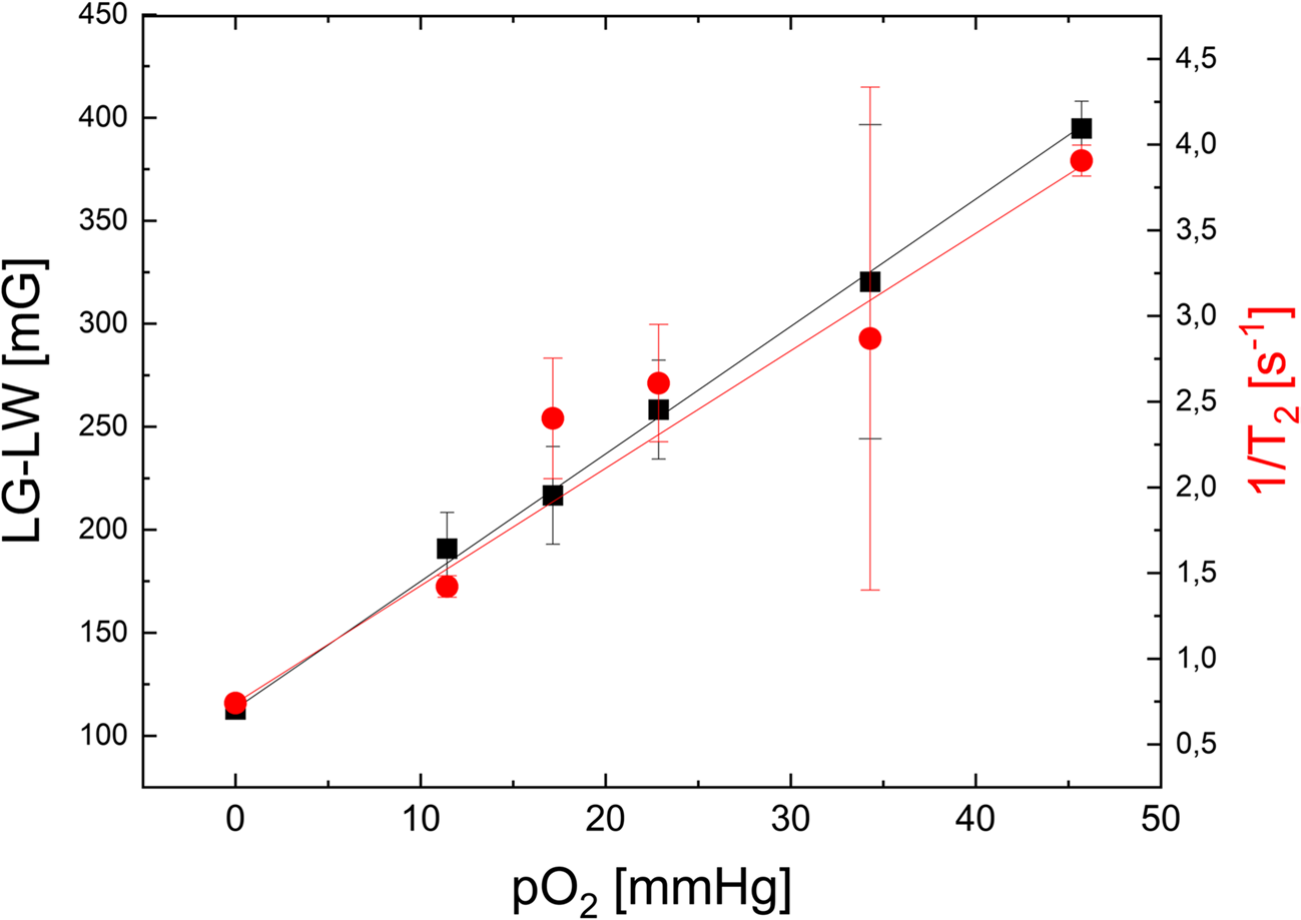
pO_2_ calibration curves for (a) black, squares: CW EPR signal fitted with Lorenzian-Gaussian function to calculate peak-to-peak linewidth (LG-LW); (b) red, circle: inverse of T_2_ relaxation time measured by pulse EPR (1/T_2_). Linear fits were performed, resulting in ΔpO_2_ = 1 torr when LG-LW changed by 6.18 mG and 1/T_2_ changed by 68.5 ms^−1^. Each point represents an average of 4–6 independent measurements with standard deviation

The calibration curves were determined in a solution equilibrated with a gas of appropriate oxygen content (Fig. 2). T_2_ was measured using pulse spectroscopy and presented as 1/T_2_ (R_2_) versus pO_2_. On the obtained calibration curve, a linear fit was performed (*r* = 0.998, *r*^2^ = 0.996). A very similar relationship was found when the Lorentzian-Gaussian function was fitted to the Oxychip CW EPR signal and the peak-to-peak signal line width (LG-LW) was calculated (*r* = 0.999, *r*^2^ = 0.999). Linear fits resulted in ΔpO_2_ = 1 torr when LG-LW changed by 6.18 mG and 1/T_2_ changed by 68.5 ms^−1^. The median SD of the measurements is approximately 3 mm Hg for both techniques. An oximetric probe of the same batch of synthesis was used in all experiments, as each batch requires its own calibration [16]. We calculated T_2_ from the Lorentzian component of the fitted CW EPR spectrum LW = 1/T_2_/gamma, gamma = 17.6 Ms-1/G), (Fig. S1). The correlation between physically measured T_2_ (Pulse EPR) and calculated from CW EPR spectrum is significant but very week (*r* = 0.2113, *p* = 0.0043, *r*^2^ = 0.0447).

### Correlation Between CW and Pulse

**Fig. 3:**
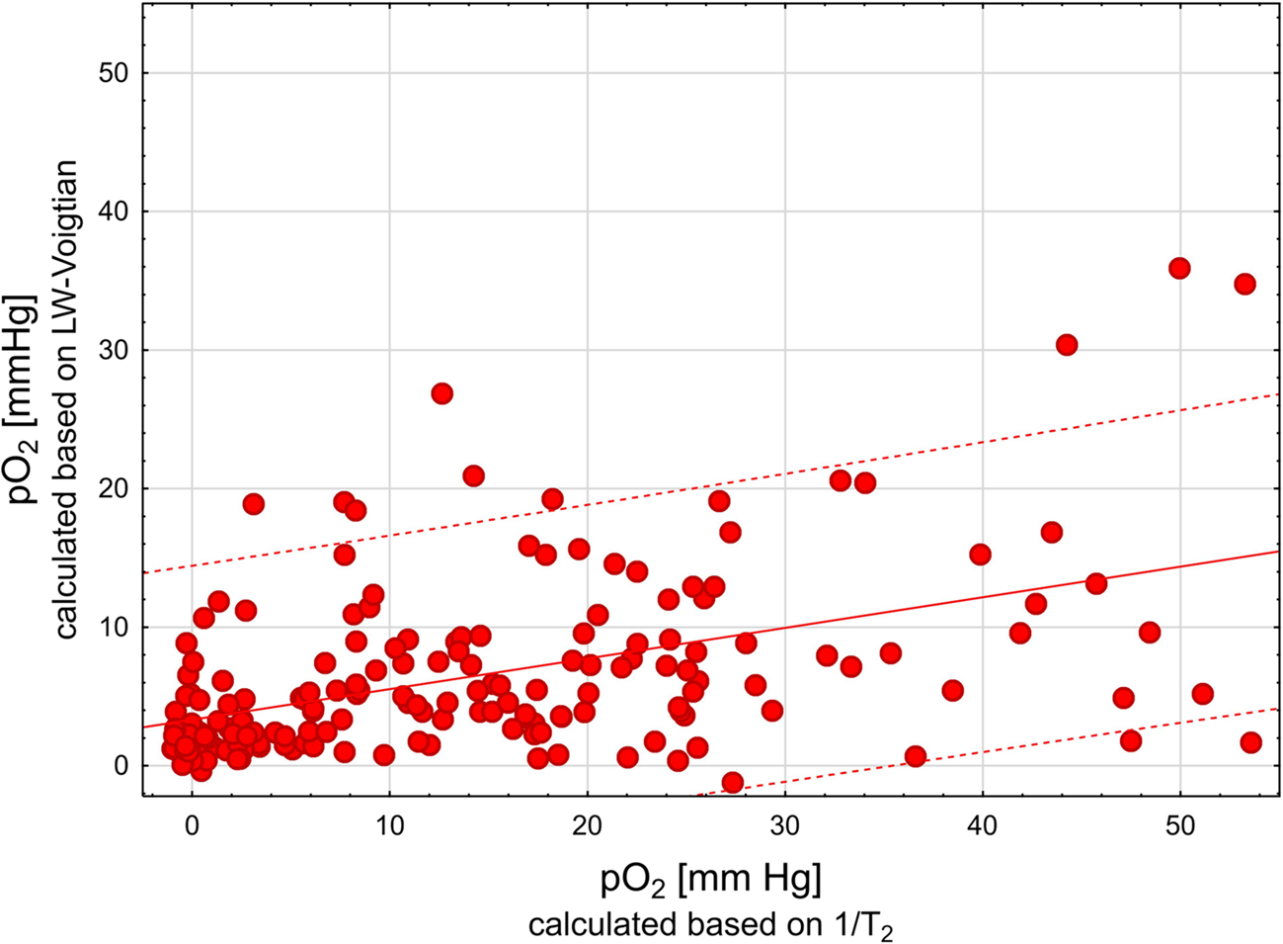
*X* axes - Pulse EPR *Y* - CW EPR pO_2_ by 1/T_2_ collected during Pulse EPR measurement (*Y* axes) and pO_2_ calculated based on LW data from CW EPR (*X* *Y* axes). The solid line represents the linear fit (*r* = 0.4405, *p* < 0.0001, *r*^2^ = 0.1941), and the dashed line represents the predictions of the 0.95 fit

During tumor therapy, Pulse EPR was used to measure the T_2_ relaxation time, while CW EPR was used to calculate the signal peak-to-peak linewidth based on the Voltgin fit to the spectrum. Animals were first measured by Pulse EPR and immediately transferred to CW EPR during the same anesthesia period. This procedure allowed comparing pO_2_ measured with both techniques (Fig. 3). However, the mean pO_2_ values obtained by CW EPR were found to be lower by approximately 4 mm Hg compared to the pO_2_ values calculated from the T_2_ measurements. The linear correlation between the pO_2_ data obtained by CW and Pulse EPR showed a weak correlation with a Pearson correlation coefficient of 0.4732 (*p* < 0.0001, *r*^2^ = 0.2239).

### Oxygenation in Pancreatic Tumors During Treatment

**Fig. 4:**
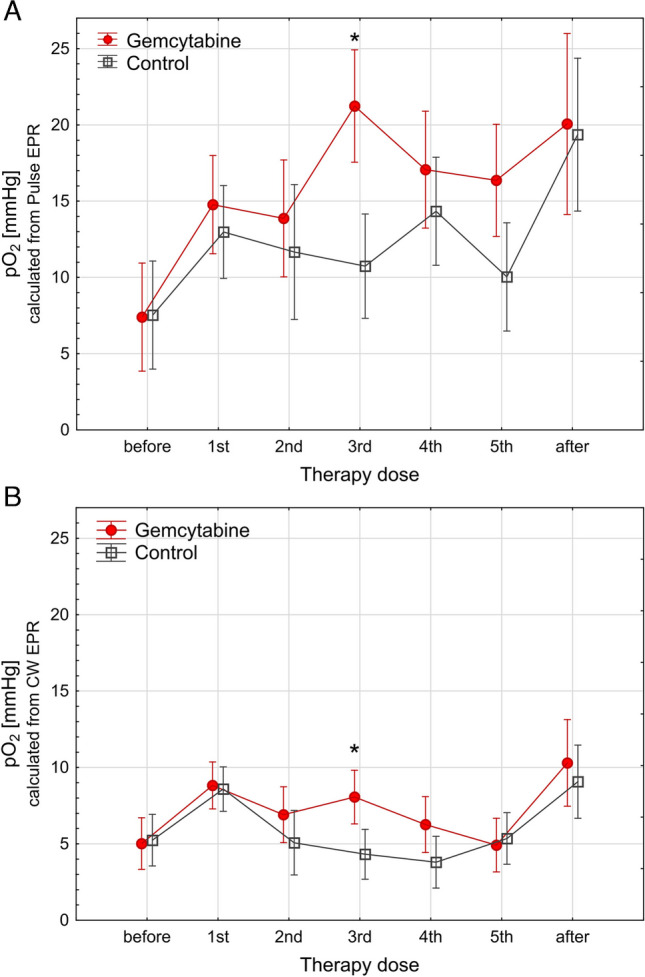

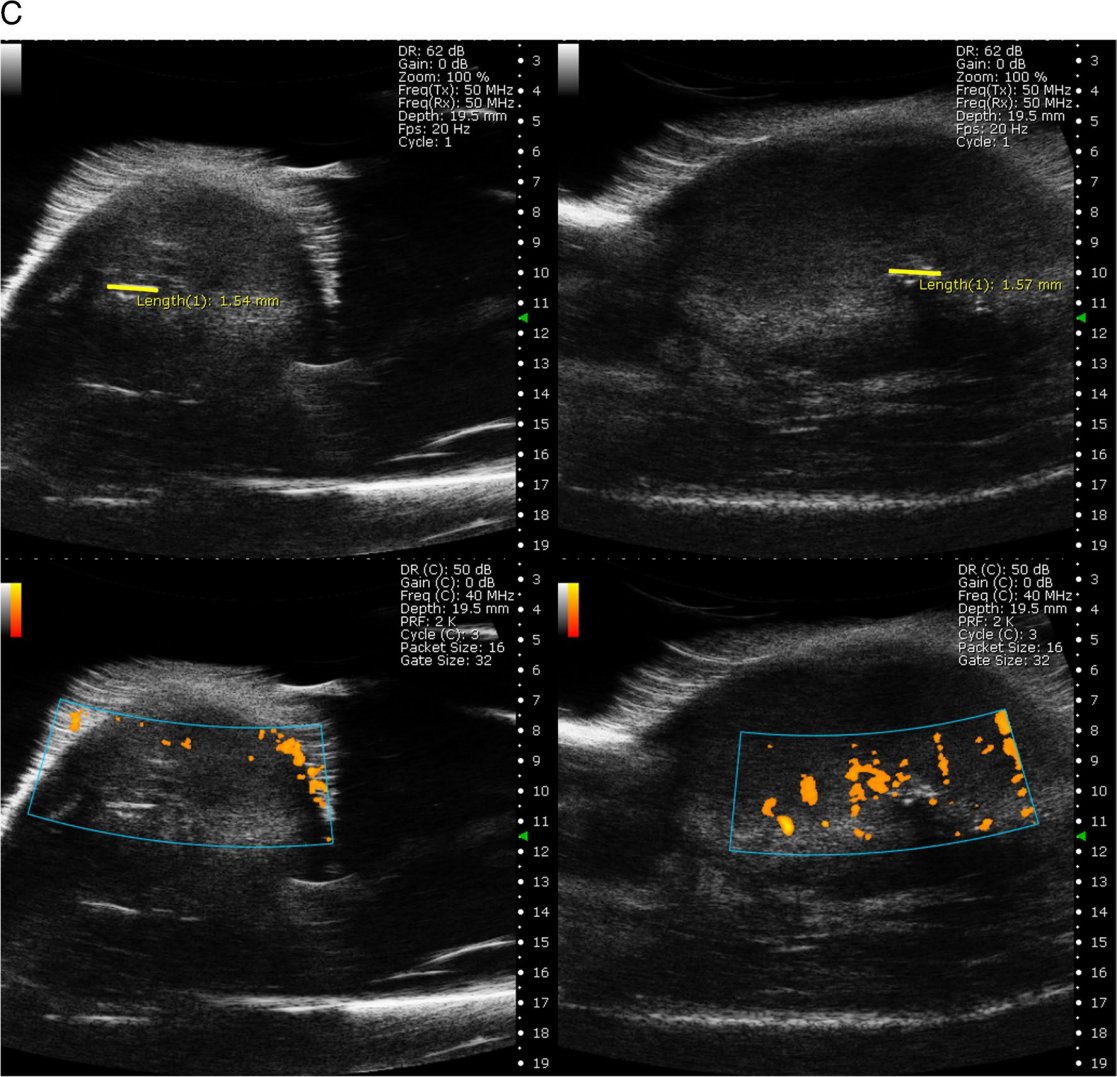
Average kinetics of pO_2_ during gemcitabine therapy. The animals received 60 mg/kg of BW gemcitabine in five doses, separated by 72 h (*N* = 14, red, circle), and the control animals received the drug vehicle at the same time points (*N* = 15, black, squares). Points represent mean with standard error of mean marked as error bar. **A** pO_2_ from murine tumors was calculated from 1/T_2_ measured by Pulse EPR. **B** pO_2_ collected from tumors measured by CW EPR and calculated based on LG-LW. * Kruskal-Wallis nonparametric statistics, *p* < 0.05. The number of animals decreased in the experimental groups due to tumor outgrowth, which is why the points after therapy represent a subpopulation of tumors characterized by slower cancer growth. **C** Representative B-mode (upper panel) ultrasound images of tumors with implemented Oxychip marked in yellow and corresponding Power Doppler measurements showing tumor vasculature around the place of probe implantation (lower panel)

EPR spectroscopic measurements were performed before treatment and several times after each dose of saline or gemcitabine (Fig. S2). The animals were measured every 2–4 days with anesthesia time of around 30–45 min. Three days after the fifth dose of therapy, the surviving animals were entering the post therapeutic phase. The pO_2_ values from 1/T_2_ pulse measurements were generally between 5 and 24 mm Hg, with SD for all measurements equal to 13.5 mm Hg (Fig. 4A). A slight increase in pO_2_ is observed in gemcitabine-treated animals after the third dose to around 21.2 ± 5.6 mmHg, while in control, the pO_2_ remained steady around 10.7 ± 3.14 mm Hg.

The pO_2_ calculated from LG-LW showed levels from −1 to 35.9 mm Hg (25–75%: 1.9–8.5 mm Hg) throughout the 21-day observation in both the control and treated group, with a SD of approximately 6.2 mm Hg (Fig. 4B). Here, the time dependence of pO_2_ was more similar in both control and treated animals. Again, significant change was observed between controls and gemcitabine-treated animals after third chemotherapy dose (test Kruskal-Wallis: *N* = 28, *p* = 0451).

An increase in pO_2_ observed after the end of therapy was measured only in a few remaining animals (*n* = 7 with tumor under control for ≥ 16 days) and therefore does not reflect the response for the whole experimental group.

The pO_2_ in tumors before treatment was 7.73 ± 7.90 mm Hg calculated from 1/T_2_ measurements and based on LG-LW data the tumor before treatment has on average 7.40 ± 5.41 mm Hg. We confirmed that tumor oxygenation and tumor volume before therapy do not influence significantly the outcome of treatment (Fig. S3).

Validation of the Oxychip location was performed by ultrasound imaging (Fig. 4C); this allows to claim that oxygen information is related to tumor tissue.

### ΔpO_2_ During Therapy as a Marker of Mouse Survival

**Fig. 5:**
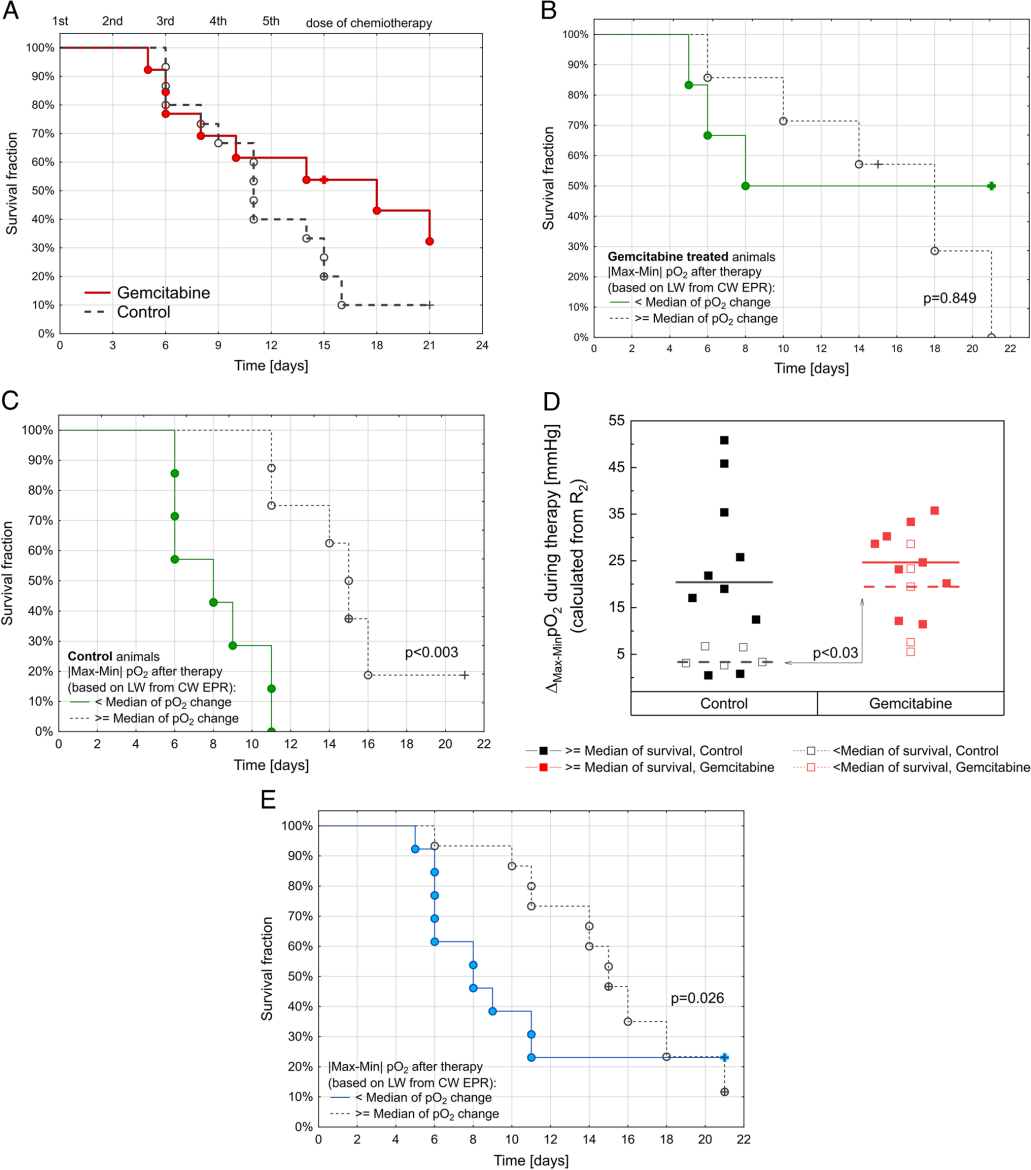
Survival analysis of (**A**) gemcitabine-treated tumors (*N* = 14) compared to controls (*N* = 15), no significant differences were detected (*p* = 0.284). To investigate the importance of pO_2_ dynamic during therapy, min and max pO_2_ were found (after the first dose of gemcitabine and 72 h after the fifth dose). Then, |max–min| pO_2_ was calculated and the median |max–min|pO_2_ change was 9.11 torr for the data collected from LG-LW. **B**, **C** Survival analysis of (**B**) gemcitabine-treated mice, *N* = 14 and (**C**) control mice, *N* = 15 with two |max–min| pO_2_ cohorts: (green) |max–min|pO_2_ < median and (gray) |max–min|pO_2_ ≥ median. A significant difference was found between the cohorts in the control animals (*p* < 0.003, Kaplan-Meier analysis). Circle symbols represent complete observation when the tumor outgrown 350 μl; in other cases, the observation was not completed (cross symbol). **D** The median survival fraction (11 days) was calculated to divide the animals into two cohorts: (days with a tumor under 350 μl) ≥ median survival (filled squares, solid line for median) and second (days with a tumor under 350 μl) < median survival (empty squares, dashed line for median). |max–min|pO_2_ calculated from 1/T_2_ was presented for gemcitabine-treated (red) and control (black) animals. A significant difference was observed for the cohort with < median survival between treated animals (*n* = 5) and control animals (*n* = 5). **E** Survival analysis of animals in relation to pO_2_ dynamic during tumor growth (*N* = 29). The median difference in |max–min|pO_2_ was determined to be 8.6 mm Hg based on the data collected from CW EPR. To conduct the survival analysis, both gemcitabine-treated and control mice were segregated into two groups according to their |max–min|pO_2_ values: |max–min|pO_2_ < 8.6 mm Hg (blue) and |max–min|pO_2_ ≥ 8.6 mm Hg (black). The Kaplan-Meier survival analysis shows a significant difference in survival between these two groups

In gemcitabine-treated tumors (*n* = 14) compared to controls (*n* = 15), no significant differences were detected in the survival analysis (*p* = 0.284, Fig. 5A). However, the gemcitabine and control animal survival curves separate after day 10, when four drug doses were already administered. To investigate the importance of pO_2_ dynamics during therapy, we determined the minimum and maximum pO_2_ during 15 days of therapy (five drug doses of drug followed by 72 h). The difference between extreme values (|max–min|pO_2_) was calculated as a factor to describe pO_2_ dynamics. The median change in |max–min|pO_2_ was found to be 8.6 mm Hg for the data collected from CW EPR. For survival analysis, gemcitabine-treated mice (Fig. 5B) and control mice (Fig. 5C) were divided into two cohorts based on |max–min|pO_2_ values: |max–min|pO_2_ < 8.6 mm Hg and |max–min|pO_2_ ≥ 8.6 mm Hg. A significant difference between cohorts was observed in control animals (*p* < 0.003, Kaplan-Meier analysis) but not in gemcitabine-treated mice. This indicates that untreated tumors with less oxygen dynamics (|max–min|pO_2_ < 8.6 mm Hg) are more aggressive and lead to shorter survival of animals. The relevance of pO_2_ dynamics (|max–min|pO_2_) indicates that this factor has a significant influence (*p* = 0.026) on tumor control and animal survival (Fig. 5E). Furthermore, the median survival time (11 days) was calculated to divide the animals into two cohorts according to tumor growth. We assumed a tumor volume of less than 350 μl as controlled. One cohort contained animals with days with a tumor under 350 μl were larger or equal to 11 days, and the second cohort contained animals with days with a tumor under 350 μl for less than 11 days. The |max–min|pO_2_ calculated from 1/T_2_ was presented for gemcitabine-treated and control animals (Fig. 5D), and a significant difference was observed for the cohort with < median survival between gemcitabine-treated (*n* = 5) and control animals (*n* = 5). Based on the presented data, it can be concluded that untreated tumors with small pO_2_ dynamic exhibit fast growth and significantly reduce the survival rate of mice. Observed significance for oxygen dynamic in tumors was noticed for |max–min|pO_2_ calculated from Pulse and CW EPR.

## Discussion

The calibration curve shows an oxygen sensitivity of approximately 6.6 mG/mmHg, similar to previously reported [9, 16]. However, some of our oxygen concentrations were produced in a gas mixer and resulted in a greater spread of acquired spectroscopic parameters (both T_2_ relaxation rate and Lorentzian-Gaussian line width). The worst reproducibility is seen with the point of 4.5% oxygen due to flowmeters that lack precision. The advantage of the CW EPR technique is its sensitivity to a wide range of oxygenation, which allows measurements of very high oxygen levels [18, 19]. The sensitivity in pulse EPR was 68.1 ms^−1^/mm Hg. The measurements based on T_2_ relaxation time are accurate, with a very good resolution and the most accurate fitting, especially for very low oxygen concentration where CW EPR does not have such sensitivity due to low spectra resolution in very narrow linewidth. However, limit of signal detection lies around 6% (45.7 mm Hg) of oxygen (when relaxation time drops below 1 μs), and higher oxygen concentrations cannot be measured using Oxychip as a spin probe with our pulse instrument. Oxychip is a LiNc-BuO in PDMS, a gas-permeable and neutral polymer. It is highly resistant to temperature, radiation, or chemical damage [20, 21]. Furthermore, in tissues, it does not generate any inflammatory response and may remain in tissue for a long time [22, 23]. A LiPc-PDMS probe was implanted for 6 weeks in mice showing good stability [24]. The difference observed between 1/T_2_ calculated from the fitted CW spectrum and measured T_2_ indicates that our model of interactions between LiNc-BuO and oxygen need more clarification.

Previous studies have shown that EPR oximetry closely reflects changes in the oxygenation of different tumor tissues depending on the applied chemotherapeutic treatment methods, which change the partial pressure of oxygen [25–27]. Significant changes in pO_2_ in tumor tissue were expected in response to gemcitabine treatment. As the number of living and metabolizing cells decreases in the tumor with the course of the therapy, an increase in pO_2_ within the tumor should be observed. In fact, in Pan02 tumors, an increase of 11 mmHg was observed after the third dose compared to the control (Fig. 4A). The dosage applied in our study induced only a mild tumor response, i.e., the survival time was increased only slightly. Gemcitabine treatment did not affect mouse survival in general; however, hypoxic tumors with low pO_2_ dynamics have the worst prognosis (Fig. 4D). The dynamic changes in pO_2_ during tumor growth are a critical determinant of animal survival (*p* = 0.026, Fig. 5E), whereas the impact of gemcitabine treatment is relatively smaller and insignificant.

The experiment utilized a subcutaneous inoculation model, in which Pan02 cells were injected into the leg. Although this model partially replicates the tumor microenvironment unique to pancreatic cancer cells, including a high density of stroma and the presence of immunosuppressive cells, it does not fully reproduce the clinical situation.

In another oximetric study using spin echo EPRI, a change in delta pO_2_ was demonstrated before and 48 h after treatment with evofosfamide in human pancreatic MIA Paca-2 tumors in mice. Evofosfamide itself is a prodrug that works best in a low oxygen environment where oxygen is reduced and the drug activated at the same time, while gemcitabine is an oxygen-independent cytotoxic drug. Both MIA Paca 2 and Su.86.86 tumors had pO_2_ approximately 13-14 mm Hg [28]. Another interesting oximetric study was performed using the human BxPc3 line, and pO_2_ in untreated tumors was between in the range of 15–20 mm Hg [29]. In general, our pO_2_ measurement agrees well with other pancreatic tumors.

There are several reasons why the correlation between the two techniques is weak (Fig. 3). First, the spectral resolution of the very narrow line in CW makes the resolution/accuracy lower in the low pO_2_ range. Conversely, in pulse EPR, the limit of detection for T_2_ is smaller than 1.4 ms in our setting, so lower relaxation times (for higher pO_2_) are beyond the detection threshold.

The main reason why the CW EPR pO_2_ data are lower than pulse EPR pO_2_ can be related to our measurement procedure where pulse EPR is always first and CW is always done second. After induction of anesthesia (first 15 min), the pulse EPR measurement took the next 15-30 min and CW EPR takes place around 30–45 min after induction of anesthesia. It is possible that the decrease of around 4 mm Hg is related to prolonged anesthesia. This hypothesis is supported by the calibration curves of pO_2_ presented in Fig. 2, where two fits (for LG-LW and 1/T_2_) have a similar slope of the curve, and no significant shift on the *X*-axis (pO_2_) was seen.

EPR oximetry is a valuable technique for quantitative assessment of pO_2_ levels in tissues *in vivo* and is minimally invasive. The ability to quantitate tumor oxygenation in the wide range of pO_2_, but especially low pO_2_ values, makes it an ideal method to monitor changes during tumor growth and after pharmacologic interventions [30, 31].

Particulate oximetric spin probes require the introduction of the probe into the examined tissue either as a solid, injection in the form of a finely ground powder suspension, or implantation together with tumor cells [29, 32–34]. The main advantages of solid probes include high spin density, leading to high SNR, high sensitivity to oxygen, and long-term stability *in vivo*. EPR oximetry based on these probes is ideal for *in vivo* measurements repeated over a long period of time; however, it should be added that the measurement of oxygen concentration is performed only from the site of probe implantation. A wide range of naturally occurring, semi-synthetic, and synthetic carbon- and lithium-based probes have been developed for EPR oxygen measurement. Some carbon derivatives have been selected with excellent spectroscopic and oxygen sensor properties, leading to their use in several clinical trials [35, 36]. Other studies demonstrated the feasibility of using India ink, a suspension containing carbon derivatives [37]. There are other clinically approved suspensions for medical marking [38].

## Conclusions

We demonstrated the feasibility of Oxychip oximetry using pulse EPR in the range between 0 and 6% of oxygen *in vivo*. The pO_2_ values of appr. 40 mmHg were the highest we were able to register under our conditions. The T_2_ relaxation times are nicely correlated with pO_2_; however, for relaxation times <1.4 μs, less accuracy was observed due to lower SNR and instrument detection range. Both CW and pulse approaches allow fast and non-invasive *in vivo* measurements (< 15 min) to obtain high-quality data. Changes in spin probe relaxation times, as well as signal linewidth (concerning the level before treatment), can possibly predict promising and unsuccessful responses to gemcitabine in hypoxic tumors, e.g., the slight change in oxygenation during gemcitabine treatment correlated with better mouse survival.

### Supplementary Information

ESM 1 **Figure S1**. The correlation of pO_2_ [mm Hg] with 1/T_2_ [s^-1^], whereas T_2_ was measured on (black squares) pulse EPR and (red circles) 1/T_2_ was calculated based on Lorenzian component of Lorenzian-Gaussian fit to CW spectras. The difference observed between 1/T_2_ calculated from the fitted CW spectrum and measured T_2_ indicates that our model of interactions between LiNc-BuO and oxygen need more clarification.(JPG 726 kb) (JPG 726 kb)

ESM 2 **Figure S2.** Histograms with the number of counts of oxygen partial pressure [mm Hg] per treatment group (gemcitabine – first row, control – second row) for specified time points in relation to the chemotherapy regiment. (**A**) Histograms based on Pulse EPR(JPG 720 kb) (JPG 720 kb)

ESM 3 **Figure S2.** (**B**) CW EPR can be used to track increased or decreased counts of hypoxic locations of Oxychip within the tumors (first column with pO_2_ <10 mm Hg).(JPG 736 kb) (JPG 736 kb)

ESM 4 **Figure 3S.** Correlation between animal survival and pO_2_ (**A**) calculated from Pulse EPR (JPG 806 kb)

ESM 5 **Figure 3S** (**B**) CW EPR. (JPG 764 kb)

ESM 6 **Figure 3S** (**C**) Animal survival correlation with tumor volume before the therapy. Linear fits with presented statistics indicate a deficiency of significant correlations between tested factors. (JPG 707 kb)
