# Supplementary figures and images for: Pulse and CW EPR Oximetry Using Oxychip in Gemcitabine-Treated Murine Pancreatic Tumors
Source: Mol Imaging Biol. 2023 Oct 2;26(3):473–83. doi: 10.1007/s11307-023-01859-w (PMC11211198; doi:10.1007/s11307-023-01859-w)

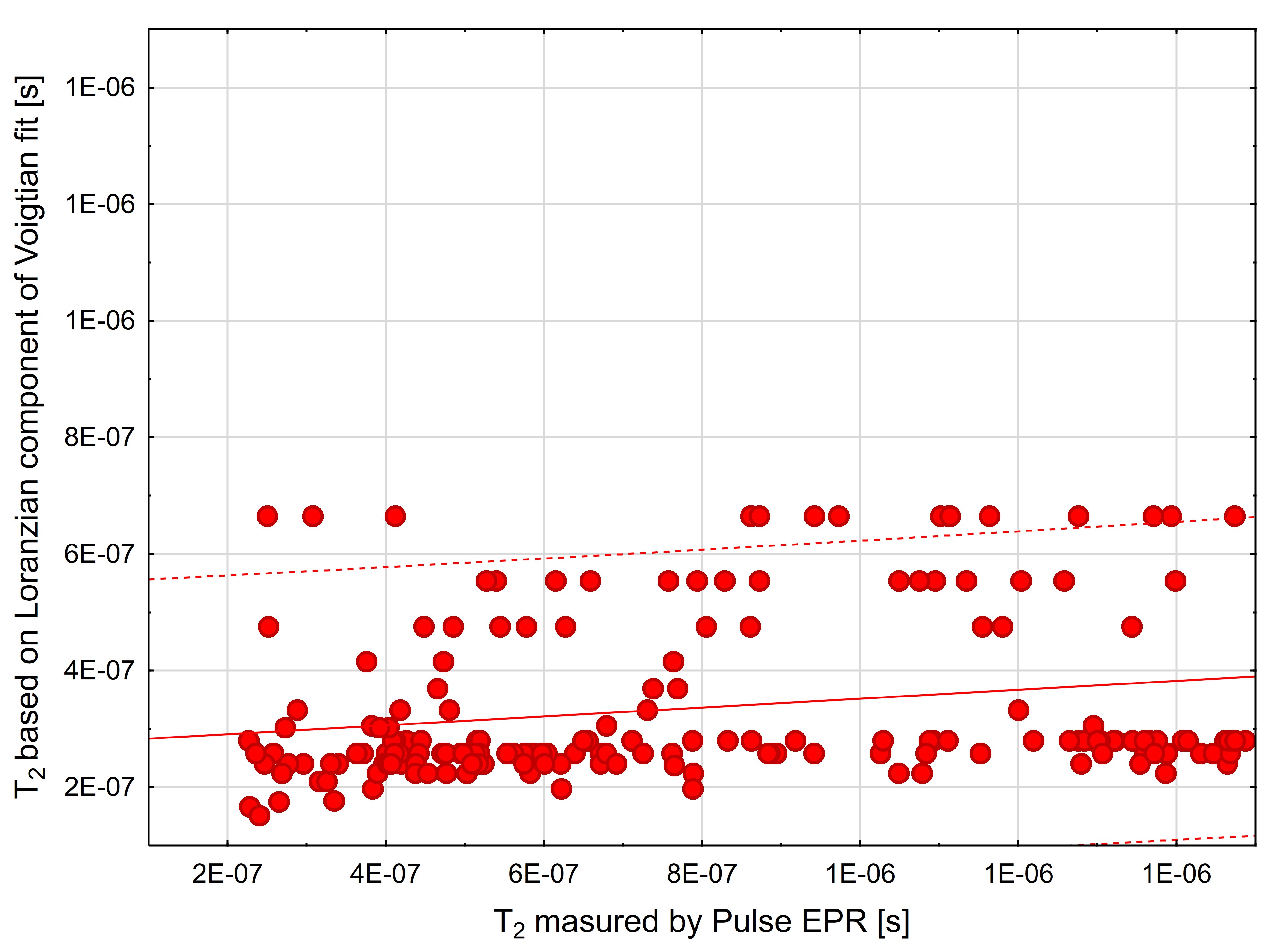

Supplement: Supplementary file 1 — Figure S1. The correlation of pO2 [mm Hg] with 1/T2 [s-1], whereas T2 was measured on (black squares) pulse EPR and (red circles) 1/T2 was calculated based on Lorenzian component of Lorenzian-Gaussian fit to CW spectras. The difference observed between 1/T2 calculated from the fitted CW spectrum and measured T2 indicates that our model of interactions between LiNc-BuO and oxygen need more clarification.(JPG 726 kb) (JPG 726 kb) [file 11307_2023_1859_MOESM1_ESM.jpg]

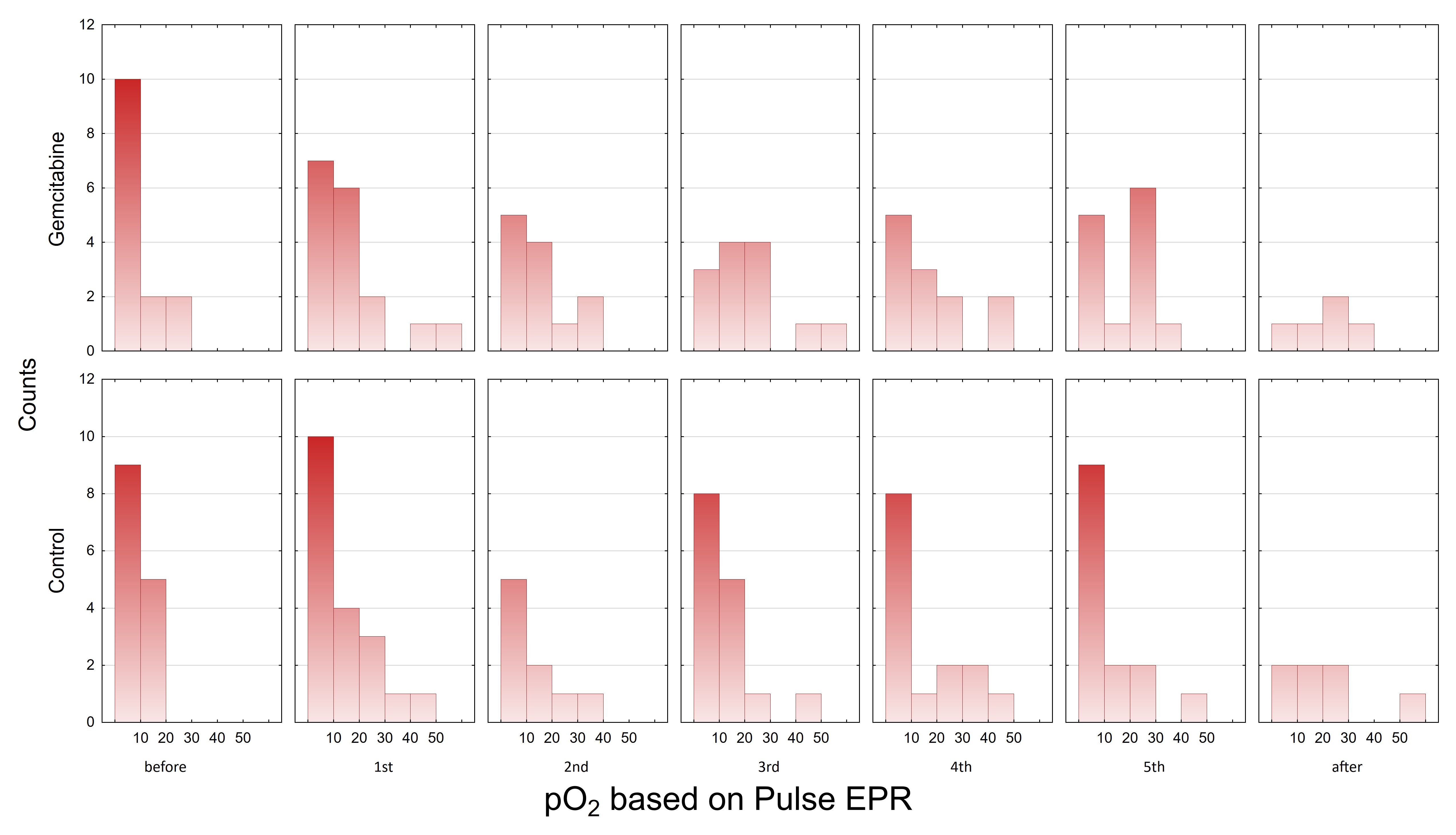

Supplement: Supplementary file 2 — Figure S2. Histograms with the number of counts of oxygen partial pressure [mm Hg] per treatment group (gemcitabine – first row, control – second row) for specified time points in relation to the chemotherapy regiment. (A) Histograms based on Pulse EPR(JPG 720 kb) (JPG 720 kb) [file 11307_2023_1859_MOESM2_ESM.jpg]

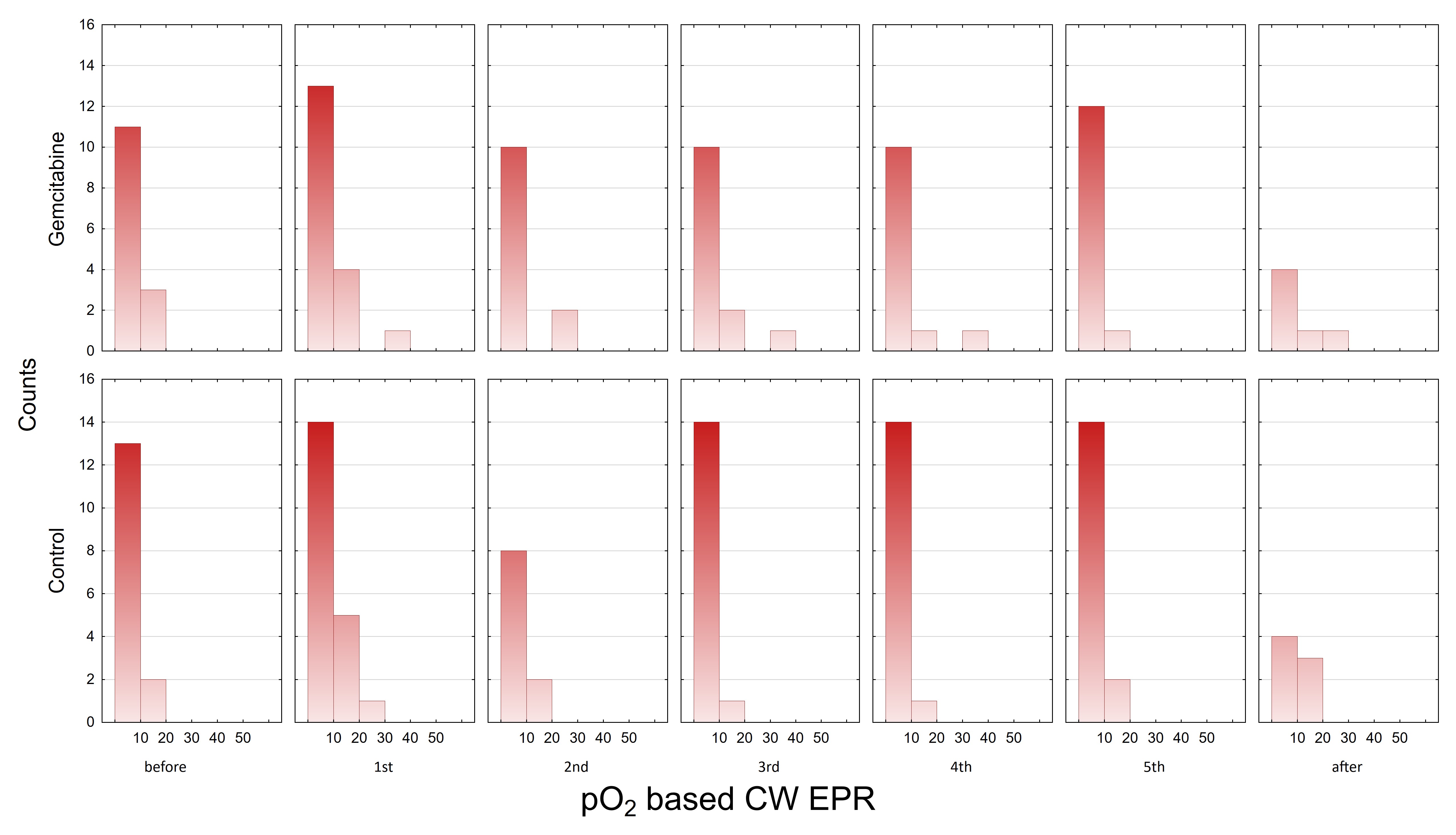

Supplement: Supplementary file 3 — Figure S2. (B) CW EPR can be used to track increased or decreased counts of hypoxic locations of Oxychip within the tumors (first column with pO2 <10 mm Hg).(JPG 736 kb) (JPG 736 kb) [file 11307_2023_1859_MOESM3_ESM.jpg]

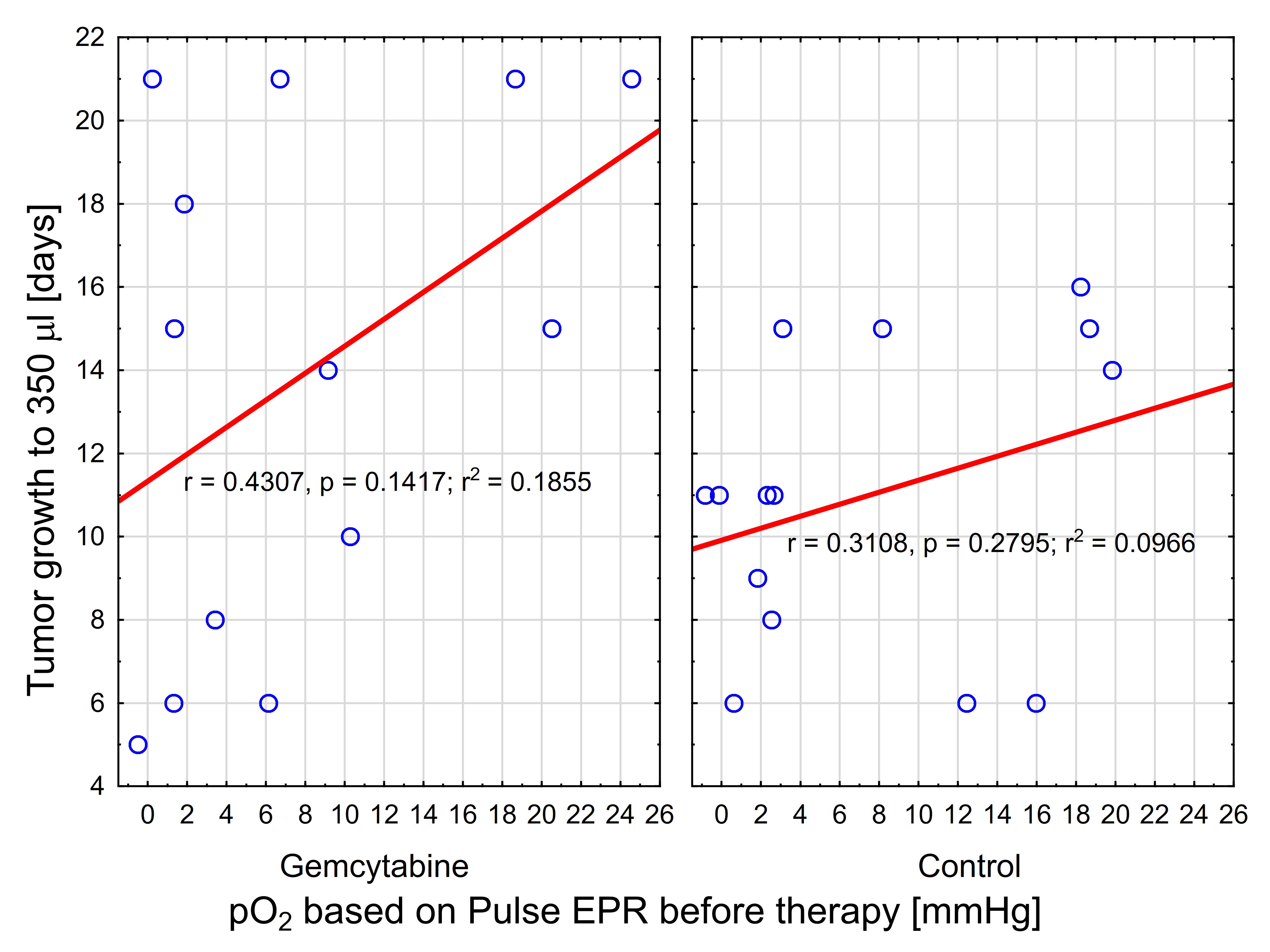

Supplement: Supplementary file 4 — Figure 3S. Correlation between animal survival and pO2 (A) calculated from Pulse EPR (JPG 806 kb) [file 11307_2023_1859_MOESM4_ESM.jpg]

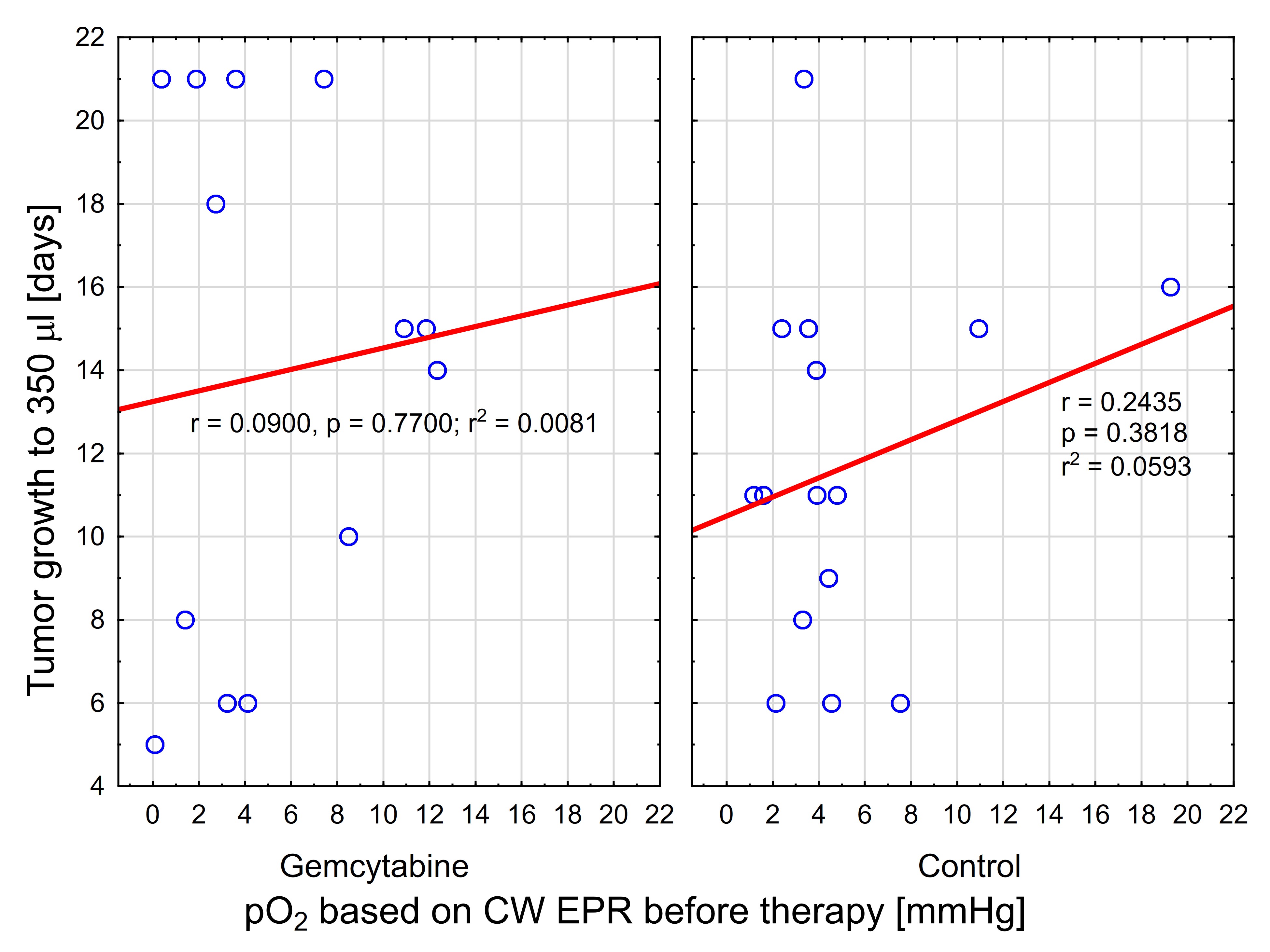

Supplement: Supplementary file 5 — Figure 3S (B) CW EPR. (JPG 764 kb) [file 11307_2023_1859_MOESM5_ESM.jpg]

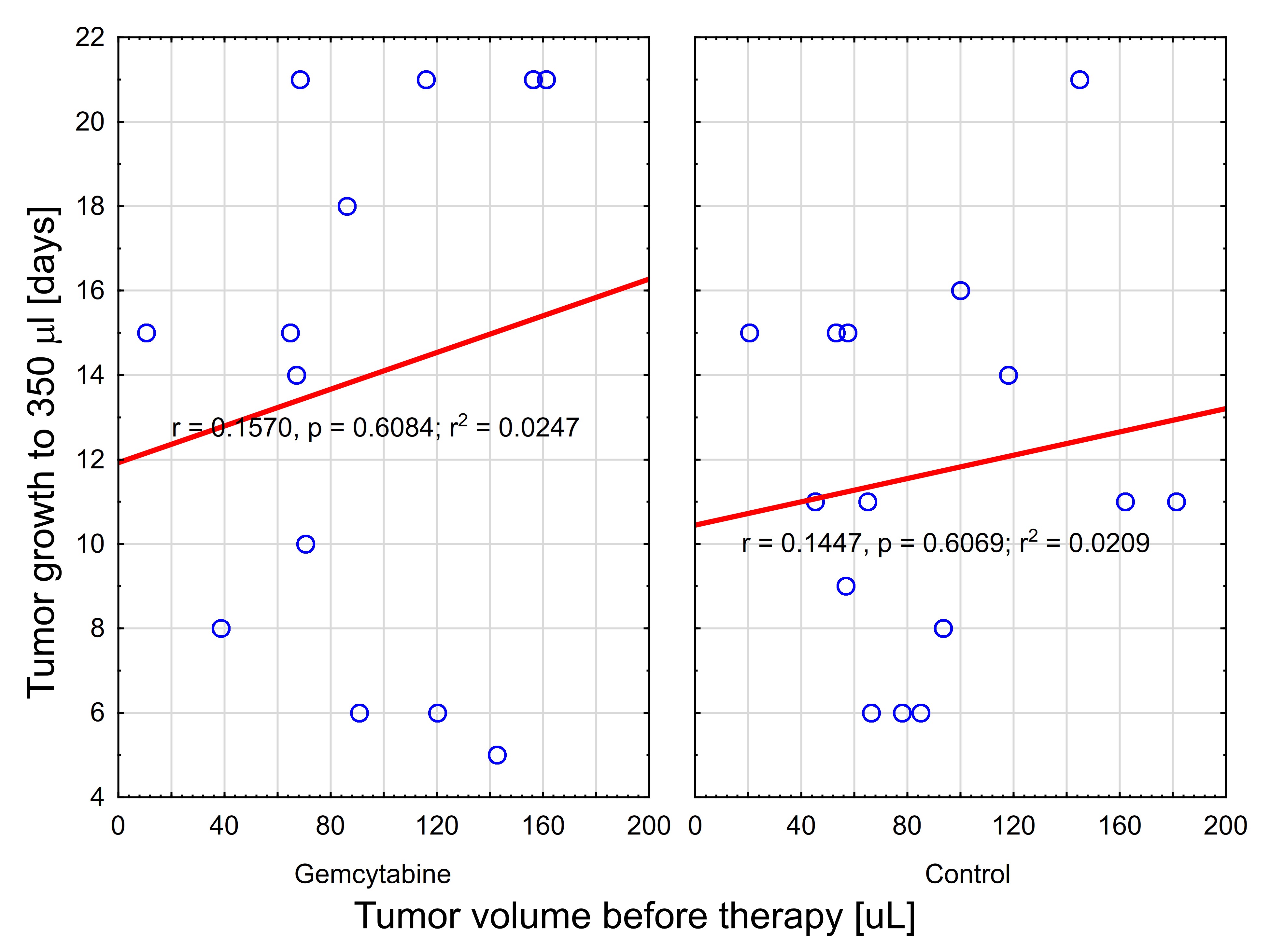

Supplement: Supplementary file 6 — Figure 3S (C) Animal survival correlation with tumor volume before the therapy. Linear fits with presented statistics indicate a deficiency of significant correlations between tested factors. (JPG 707 kb) [file 11307_2023_1859_MOESM6_ESM.jpg]
